# A Modular 512-Channel Neural Signal Acquisition ASIC for High-Density 4096 Channel Electrophysiology †

**DOI:** 10.3390/s24123986

**Published:** 2024-06-19

**Authors:** Aikaterini Papadopoulou, John Hermiz, Carl Grace, Peter Denes

**Affiliations:** Lawrence Berkeley National Laboratory, Berkeley, CA 94720, USA; jthermiz@gmail.com (J.H.); crgrace@lbl.gov (C.G.); pdenes@lbl.gov (P.D.)

**Keywords:** brain–machine interface, biomedical electronics, in vivo, high-channel count, neural readout, biopotential recording, front-end circuits

## Abstract

The complexity of information processing in the brain requires the development of technologies that can provide spatial and temporal resolution by means of dense electrode arrays paired with high-channel-count signal acquisition electronics. In this work, we present an ultra-low noise modular 512-channel neural recording circuit that is scalable to up to 4096 simultaneously recording channels. The neural readout application-specific integrated circuit (ASIC) uses a dense 8.2 mm × 6.8 mm 2D layout to enable high-channel count, creating an ultra-light 350 mg flexible module. The module can be deployed on headstages for small animals like rodents and songbirds, and it can be integrated with a variety of electrode arrays. The chip was fabricated in a TSMC 0.18 µm 1.8 V CMOS technology and dissipates a total of 125 mW. Each DC-coupled channel features a gain and bandwidth programmable analog front-end along with 14 b analog-to-digital conversion at speeds up to 30 kS/s. Additionally, each front-end includes programmable electrode plating and electrode impedance measurement capability. We present both standalone and in vivo measurements results, demonstrating the readout of spikes and field potentials that are modulated by a sensory input.

## 1. Introduction

The brain is perhaps the most complex system we know of; multiple brain regions contribute to any given function through complex, anatomically distributed sub-circuits. We know that neurons generate electrical activity by means of action potentials which encode information, and that the timescales of brain activity range from milliseconds to years. However, the exact way that spiking patterns encode information is still a mystery.

As the neuroscience community attempts to translate these signals, the need for large-scale, high-density neural recording increases [1]. A large number of recording sites featuring high anatomical spatial coverage and millisecond temporal resolution is necessary for any new technology developed to tackle this problem. As a result, significant progress has been made in increasing the number of electrodes in silicon and polymer probes [2,3], which in turn increases the requirement for high-channel-count neural readout electronics.

One of the most widely adopted commercial ASICs for neural readout features up to 64 recording channels [4,5]. Each channel has an AC-coupled front-end and offers ultra-low noise recording. The large capacitors required for a 1 Hz high-pass cutoff limit the scalability of the system, however, making it impractical for recording thousands of channels.

In [6,7], up to 384 readout channels are demonstrated on a single chip. The system is monolithically fabricated with electrodes and circuits on a silicon substrate and achieves a small area and very low-power recording at a moderate analog-to-digital converter (ADC) resolution. In addition, the probes can be used in multi-module assemblies of thousands of channels. Although monolithic fabrication allows increased channel count, the readout cannot be integrated with other probes or electrode technologies.

A massive 65,536-channel count recording system is demonstrated in [8]. The system consists of microwire electrode arrays bonded to readout electronics, and it is the largest recording array to date. The readout ASIC does not include digitization, and power consumption can become a serious bottleneck since even a small temperature increase at the recording site can affect the measured potentials. Furthermore, the device weight and size are too large to be used in awake and free-behaving experiments with small animals like rats.

This work achieves the readout and digitization of 512 channels onto a single chip [9]. The chip can be used in multi-module assemblies of up to eight modules, therefore increasing the channel count to 4096. The prototype borrows a 2D layout approach that has previously led to major developments in particle physics and X-ray microscopy, allowing a much higher density of electronics than standard 1D layouts. It features a DC-coupled programmable analog front-end and in-pixel ultra-low noise 14-bit digitization, as well as programmable clock distribution, and data encoding and serialization, making it a complete high-density neural readout solution compatible with various high density electrode arrays in standalone or multi-module configuration.

This work is organized as follows. First, a brief overview of the complete neural aquisition system is presented in Section 2.1 in order to provide the context for the specific ASIC. This overview is followed by an extensive section on the circuit design details for each channel in Section 2.2 before moving on to bench and in vivo measurement results in Section 3.

## 2. Materials and Methods

### 2.1. System Overview

**Figure 1:**
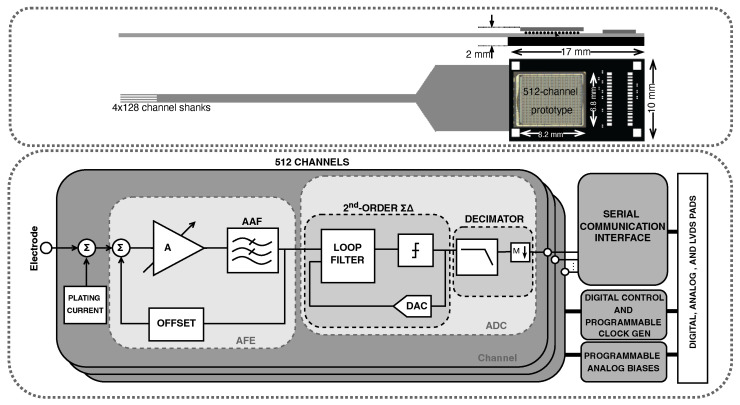
High-level system architecture of the proposed neural recording system (**top**) and readout ASIC (**bottom**). (**Top**): One module of the system consists of a flexible ribbon cable which connects four electrode shanks with the substrate. The 512-channel ASIC is bump-bonded onto the substrate, which is bump-bonded onto the stiffened ribbon cable. (**Bottom**): Block diagram of the proposed readout ASIC. The electrode signals are amplified, digitized, and encoded before being sent to the next processing stage.

The proposed ASIC is designed as the basis of a modular, high-channel-count recording system, the architecture of which is shown in the top part of Figure 1. In this target system, each module includes a 512-channel polymer probe consisting of 4 shanks of 128 electrodes, which is connected to the chip through a flexible ribbon cable. The chip is bump-bonded on a 10 mm × 17 mm substrate, which is then bump-bonded to the ribbon cable. The chip I/O is routed to an ultralight 0.35 mm pitch connector to be sent to an FPGA for processing. The weight budget is 350 mg for each module in order to allow for up to 8 modules to be stacked together inside the headstage. As a result, a total of 4096 electrodes can be simultaneously processed by a single FPGA.

### 2.2. Circuit Design

The focus of this work is the neural signal acquisition ASIC, which is shown in the bottom part of Figure 1. Each channel consists of a front-end neural amplifier with programmable gain, a programmable anti-aliasing filter, a buffer, and a 14b ΣΔ analog-to-digital converter, including the digital decimation filter. Analog biasing is provided through programmable digital-to-analog converters (DACs). Digital control, programmable clock generation and distribution, as well as a serial communication protocol are implemented on chip. Low-voltage differential signaling (LVDS) is implemented for all high-speed inputs/outputs (I/Os).

#### 2.2.1. Analog Front-End

**Figure 2:**
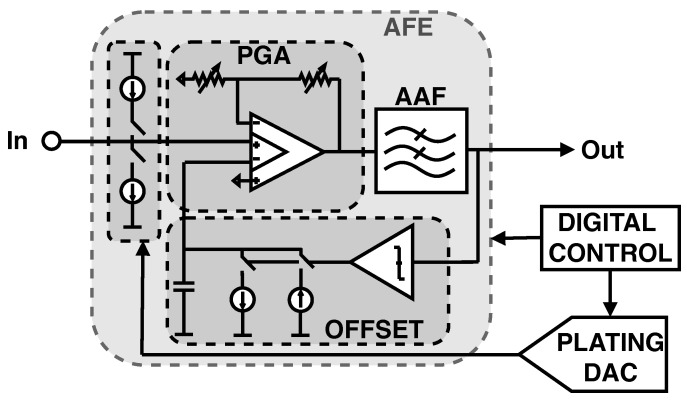
Detailed block diagram of the analog front-end, including the electrode plating current scheme.

**Figure 3:**
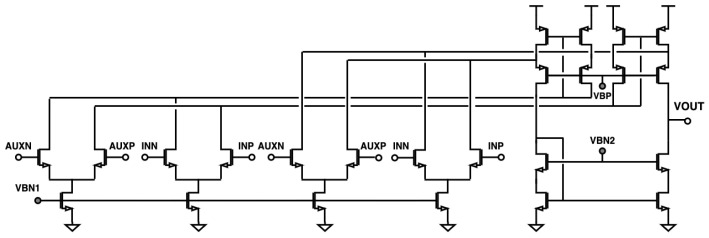
Schematic of the 4-input operational transconductance amplifier used in the analog front-end.

A detailed block diagram of the analog front-end (AFE) is shown in Figure 2. In order to achieve both a programmable gain and the ultra-high input impedance required for neural recording, the first stage of the AFE is implemented as a 4-input operational transconductance amplifier (OTA), consisting of one main and one auxiliary input pair. The topology is based on the conventional current-mirror OTA, with an additional current-stirring input pair as well as cascoded biasing devices to further increase the achievable gain (Figure 3). The main amplifier has high-input impedance and is DC coupled to the electrode pad. DC coupling has the advantage of much lower area than AC coupling, but it is more sensitive to electrochemical offsets.

In order to compensate for electrochemical offsets while maintaining power, area, and noise requirements, a simple background offset calibration is implemented as feedback to the main amplifier; if the amplifier output exceeds a programmable threshold, a current charges a capacitor connected to the inverting input so that the amplifier output voltage is zero. This large capacitor is implemented as a MOS capacitor (MOSCAP) to minimize area while maintaining good charge retention. The offset correction scheme is implemented offline and does not otherwise interfere with the signal path. The auxiliary inputs of the amplifier are used to set the gain through an externally programmable resistive ladder. The available values for the amplifier gain are 5, 10, 20, 50, 100, and 200. The final stage of the AFE is an anti-aliasing filter (AAF) with a programmable cutoff frequency.

In addition to gain, filtering, and offset calibration, the AFE features an electrode plating capability. Electrode plating currents are provided through gated current mirrors. A DAC common to all channels sets the value of the plating current. Each pixel can be individually programmed to source or sink the plating current. The electroplating feature can also be used for electrode impedance measurements. Both the electrode plating process and the impedance measurement process are discussed in more detail in Section 3.1.3.

#### 2.2.2. Sigma–Delta ADC Specification

**Figure 4:**
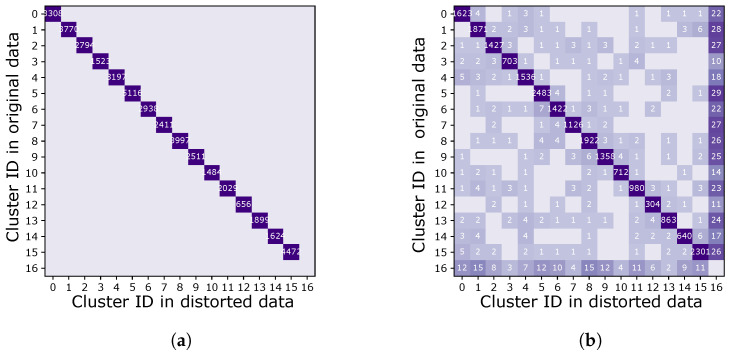
Simplified examples of confusion matrices of two data sets of spike data. The number in each square represents the number of events in each cluster. Off-diagonal events are either missed or misidentified events. In (**a**), the confusion matrix shows no information loss between the two data sets. In (**b**), the confusion matrix shows both missing and misidentified events.

To optimally determine the ADC specifications, a spike-sorting algorithm was applied to publicly available 16-channel rat neural recordings, which were reconstructed to create a golden data set. The golden data were then digitized in software with various levels of non-idealities, including quantization, non-linearity, and noise. The golden data as well as the non-ideal data were processed through a spike-sorting algorithm that produces neural clusters [10]. The results were visualized using a confusion matrix (Figure 4). Events that appear on the diagonal on the matrix are correctly matched between the two data sets, whereas off-diagonal events represent either missed or misidentified events. Additional analyses that explore spike-sorting accuracy as a function of ADC specifications can be found in [11]. This process enables setting system specifications that are informed by spike-clustering algorithms.

It was a determined that 14-bit quantization causes a sufficiently small error in neural clustering produced by spike sorting. Linearity requirements are relaxed, and noise requirements allow the effective number of bits of the ADC to be as low as 12 bits. In addition, bandwidth requirements are limited to less than ≤10 kHz due to the nature of intracortical signals.

#### 2.2.3. Sigma-Delta Modulator

In order to achieve the desired specifications while maintaining low power and area, a 14-bit ΣΔ topology was chosen. ΣΔ topologies have been increasingly attractive in the field of neural recording [12,13], because they are uniquely appropriate for high-resolution, ultra-low power digitization, and they offer advantages such as quantization noise shaping and relaxed filtering requirements.

In this work, a 2nd-order ΣΔ loop with a double-sampling front-end was designed in order to provide both good stability and flicker noise suppression at a reasonable oversampling ratio. The oversampling ratio (OSR) was set to 256 to achieve a good tradeoff between required clock frequency and capacitor area such that the modulator is small enough to be integrated into the pixel. With this oversampling ratio and a desired signal sampling frequency of fs=30 kS/s, the clock speed of the modulator is $f_{clk}=OSR\times f_{s}=7.68$ MHz.

**Figure 5:**
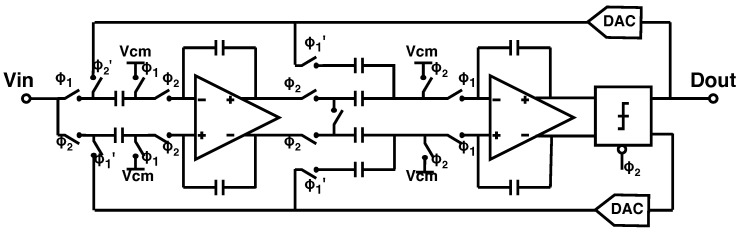
Simplified schematic of the double−sampling ΣΔ modulator topology.

**Figure 6:**
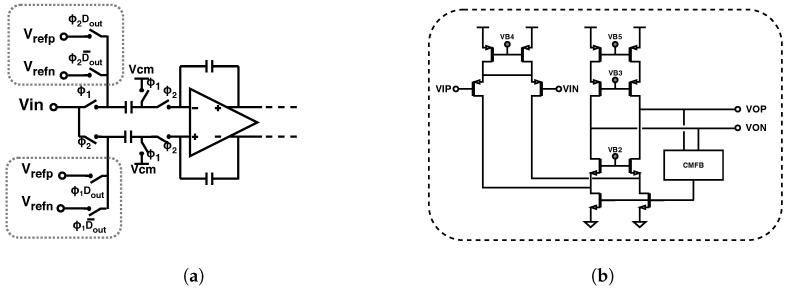
(**a**) The first integrator stage shown with more detail on the DAC implementation and references. (**b**) Schematic of the fully differential folded−cascode amplifier used in the modulator.

The modulator was implemented as a discrete-time, switched-capacitor topology, in a single-ended-to-differential configuration, as shown in Figure 5. In this topology, the input is sampled in both phases of the clock—on the top input capacitor during $\phi_{1}$ and on the bottom input capacitor during $\phi_{2}$. During the integration stage, the two samples are averaged. This double-sampling input scheme therefore implements signal averaging at every step, which relaxes the noise requirements and therefore allows for a smaller input capacitor size [14]. As a result, it is possible to maintain reasonable linearity while maximizing dynamic range. A more detailed schematic of the DAC implementation is shown in Figure 6a. In each phase of the clock, the input capacitors are either shorted to the input or to one of the references, depending on the output of the modulator. The signals that drive the DAC switches are implemented using standard-cell logic gates. The amplifiers were implemented using a conventional fully differential folded-cascode topology with capacitive common-mode feedback (Figure 6b). The modulator draws a total of 60 μA when the ADC is operated at the maximum sampling frequency of 30 kS/s, including the input buffer current.

#### 2.2.4. Digital Decimation Filter

In addition to a low-noise analog front-end and a ΣΔ modulator, each pixel contains a digital decimation filter to convert the single-bit data stream from the ΣΔ modulator into a slower, multi-bit ADC output with improved resolution. The key objectives in the design of the decimation filter are minimum area, in order to fit the filter into a small pixel, and low power, to enable the inclusion of many pixels per chip.

**Figure 7:**
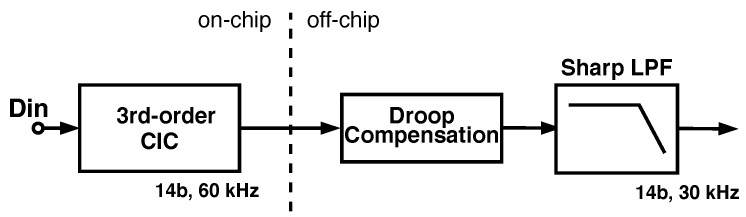
Block diagram of the proposed split decimator, which allows a tradeoff between on-chip filter area and data communication bandwidth. The output rate of the on-chip CIC filter is 60 kHz. Droop compensation as well as low-pass filtering are implemented off-chip in order to achieve the desired 30 kS/s sampling rate.

Typically, the decimation filter for a ΣΔ ADC is implemented using multiple stages, and the final stage is usually a high-order finite impulse response (FIR) filter to reject close-in aliasing due to the decimation process. This final stage often drives the area and power of the decimation filter [15]. For this ADC, we separated the decimation into two components: a pre-filter to be implemented in the pixel and a post-filter implemented off-chip, as shown in Figure 7. This partitioning allowed a tradeoff between on-chip filter area and data communication bandwidth requirements, because as the modulated signal is decimated in each stage, bandwidth is traded for resolution. Partitioning the filter between on-chip hardware and off-chip hardware or software allows the decimation filter to be optimized for the specific application, both reducing area and improving performance. Integrating the decimation pre-filter on the chip reduces the data volume that must be transmitted from the prototype by a factor of 9 compared to transmitting the raw modulator data stream.

**Figure 8:**
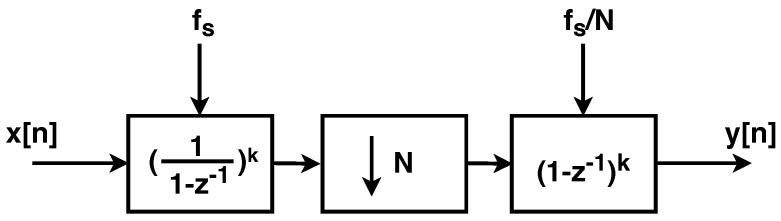
Block diagram of Cascaded Integrator Comb filter. The output bits of the ΣΔ modulator are integrated k times, downsampled by a factor of N, and then differentiated an additional k times. This structure is highly computationally efficient, as it allows the implementation of a high−order low−pass filter without multipliers, reducing power dissipation and area.

To minimize the on-chip area, the decimation filter is implemented as a Cascaded Integrator Comb (CIC) filter. This filter structure is a computationally efficient implementation of a narrow-band FIR low-pass filter that does not require multipliers, which greatly relaxes the area and power dissipation required to implement the CIC filter [16]. The implementation of the CIC filter is shown in Figure 8.

One consequence of using a CIC filter is that it has a strong sinc(x) response, which requires a droop compensation filter to recover the frequency response near the Nyquist band. Here, however, the spike-sorting routines that post-process the data acquired by the prototype include sharp low-pass filtering as part of their operations, so good high-frequency fidelity is not required in the decimation filter.

**Figure 9:**
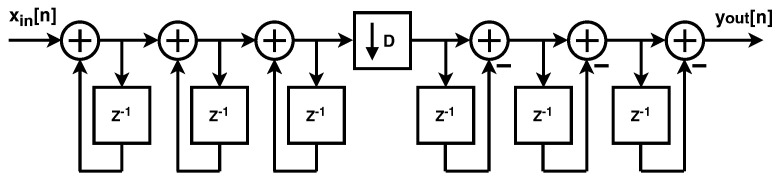
Implementation of the third order CIC filter. The filter consists of a third-order integrator, which is followed by a downsampler and a third−order differentiator. A two’s complement encoder precedes the filter.

To balance performance and complexity, typically, a CIC filter used in a ΣΔ ADC is implemented using an order that is one larger than the order of the modulator [15]. Since the ADC is using a second-order modulator, the implemented CIC is a third-order FIR filter. The CIC filter implemented as part of the ΣΔ ADC is shown in Figure 9. The filter uses a two’s complement data representation to simplify data flow, and it consists of a third-order integrator, which is followed by a downsampler and a third-order differentiator.

To ensure the final filter output does not overflow, the word width must be greater than

(1) $$W=Nlog_{2}\left(D\right)+1$$

where *W* is the required word width to avoid overflow, *N* is the order of the CIC filter, and *D* is the decimation factor. In this case, the decimation factor for the pre-filter is 128. This leads to a required word width of 22 bits [16] in the prototype CIC filter. It is possible to shrink the word width as data progresses down the pipeline, but this was not completed here, because we determined that the possible reduction in area was small relative to the additional effort required.

**Figure 10:**
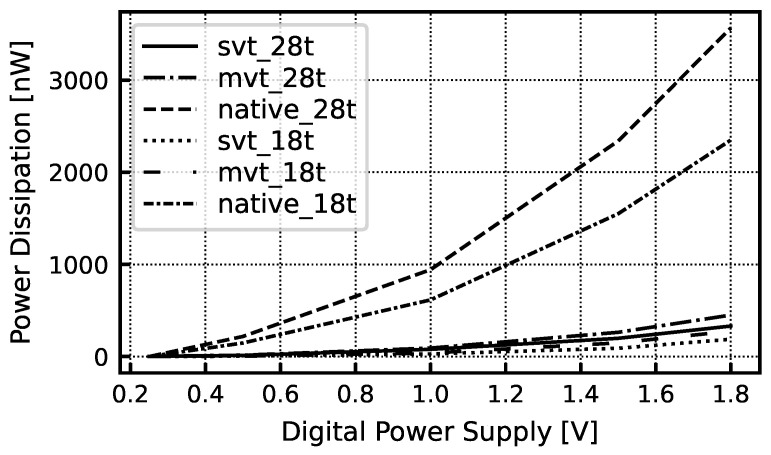
Comparison of power dissipation of several full adder topologies simulated at 4 MHz and implemented using various transistor flavors. The conventional 28-transistor full adder cell implemented using standard-threshold devices had the best balance between power dissipation, die area, and reliability across power, voltage, and temperature variation. “svt” refers to standard-threshold devices, “mvt” references to medium-threshold devices, and “native” refers to zero-threshold devices.

**Figure 11:**
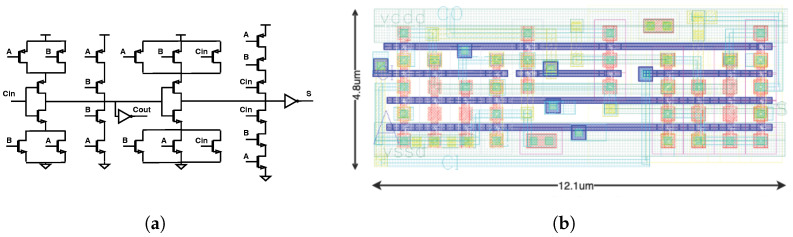
(**a**) The conventional 28-transistor full adder topology. (**b**) Custom layout of the full adder consumes 30% less die area compared to the full adder included in a commercial standard cell library.

The key goal of the decimation filter is to minimize the area to enable the integration of a complete ADC inside each pixel. Because the full adder is the key circuit in the CIC filter, a number of full adder topologies were investigated to optimize area and power dissipation. We compared a conventional 28-transistor full adder and a more aggressive 18-transistor adder based on pass-transistor logic [17]. Each full adder was implemented using transistors with various thresholds. We also examined adders with even fewer devices but found the performance variation across corners was problematic. The results of this simulation study are summarized in Figure 10. We determined that the conventional 28-transistor full adder cell (Figure 11a) implemented using standard-threshold devices had the best balance of low power dissipation, small die area, and high reliability across corners given the expected workload.

To minimize the area of the digital filter, the layout was custom designed. The dimensions of the custom full adder, shown in Figure 11b, are 12.1 µm by 4.8 µm (59.1 µm^2^), and the full adder area was reduced by over 30% compared to a commercial standard cell full adder. The area of the other cells in the filter was reduced by a similar factor. In addition, because of the use of minimum-size devices throughout the layout (to minimize area), the power dissipation of the custom full adder was reduced by a factor of approximately 3 compared to a commercial standard cell full adder.

In addition to full adders, digital latches are required to pipeline the data as it flows through the filter. To minimize area, the latches are implemented using 2-phase logic. This is possible to accomplish in a simple way by reusing the 2-phase clocks required for the switched-capacitor circuits in the ΣΔ modulator. The integrators operate at the same speed as the modulator, while the differentiators (after the downsampler) operate at 1/128th the rate of the modulator. This implicit clock division is implemented by masking every 128th phase of the higher-frequency clock.

The entire filter is implemented using a full-custom layout style without including any standard cells in the design. The filter has an area of approximately 200 µm by 100 µm or 0.02 mm^2^.

The physical implementation of the CIC filter in each pixel required 5850 transistors. The breakdown of the per-pixel CIC filter device usage is shown in Table 1. The coder block converts the single-bit output of the ΣΔ modulator into a 22-bit two’s complement representation.

#### 2.2.5. Digital Services

Two 14-bit words, representing the sample from two channels, are encoded in a 32-bit DC-balanced output word. The chip serializes the data output on two LVDS channels, each carrying the data from 16 rows × 16 columns of pixels, at a rate of 32 × 128 $\times \;f_{s}$, where $f_{s}$ is the sampling frequency. Chip programming is performed via a 2-wire interface with token passing to control multiple chips in multi-module assemblies.

### 2.3. Chip Layout and Fabrication

**Figure 12:**
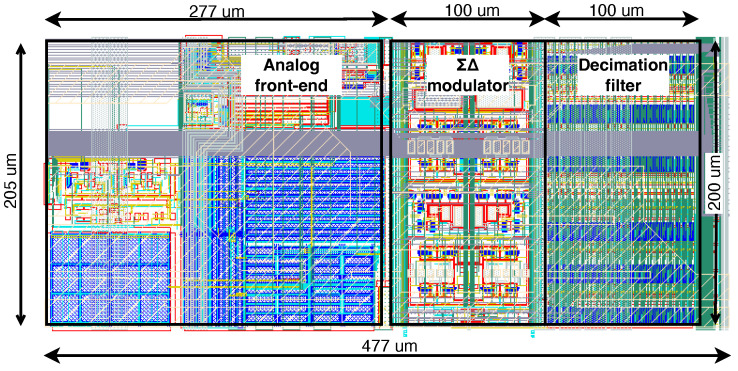
Layout of the complete pixel. The dimensions of the pixel are approximately 205 µm × 477 µm with more than half of the area dedicated to the analog-front-end. The modulator and decimation filter occupy approximately 200 µm × 100 µm or 0.02 mm^2^ each.

The chip was designed using Cadence tools and fabricated in a TSMC 0.18 µm 1.8-V CMOS process. Each channel occupies 0.099 mm^2^, including the area of the power pads. The channel layout is shown in Figure 12.

**Figure 13:**
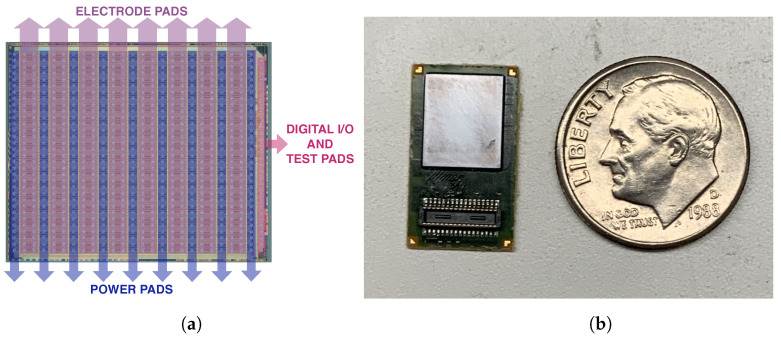
(**a**) Die photo of the chip. The die size is 8.2 mm × 6.8 mm. Power pad columns (blue) are placed between every two electrode pad columns. Digital I/O and test pads are the right-most column. (**b**) Chip prototype bump-bonded on the substrate board.

Figure 13a shows a microphotograph of the prototype along with the floorplan and power distribution scheme. Each chip contains 512 channels, which were organized as 32 rows by 16 columns. Each channel includes the electrode pad, and one column of additional power pads is placed between every two electrode pad columns. These additional power pads achieve ultra-low resistance power routing, easy decoupling, and reduced on-chip power regulation requirements at the cost of chip area.

Figure 13b shows the chip bump-bonded on the substrate board. The total chip area is 55.8 mm^2^.

## 3. Results and Discussion

Both standalone bench testing and in vivo testing were performed, and the results are presented in Section 3.1 and Section 3.2, respectively.

### 3.1. Standalone Measurements

#### 3.1.1. Chip Performance

**Figure 14:**
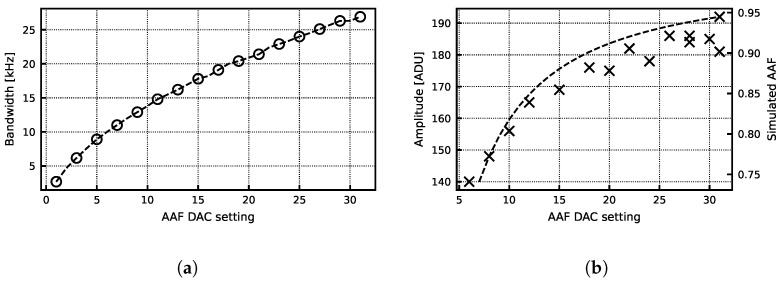
(**a**) Measured system bandwidth vs. AAF DAC value. (**b**) Simulated amplitude for a 0.2 mV 10 kHz input sinusoid compared to simulated AAF response. The AFE gain setting is 50. ADU size is 61 µV.

For standalone testing, the peripheral power pads and electrodes are wirebonded onto a custom testboard. Initial testing was completed using the programmability and testability features of the chip. Figure 14a shows the excellent bandwidth programmability of the AAF filter. Figure 14b shows the measured signal amplitude compared against the simulated AAF response for various AAF DAC settings. For an AAF DAC value of 20, the measured gain is 53.4, which is very close to the expected value.

**Figure 15:**
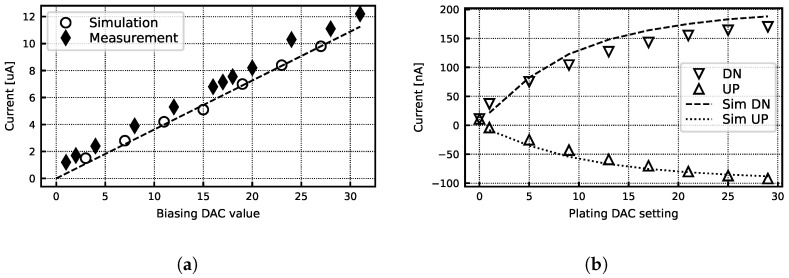
(**a**) Measured bias current vs. biasing DAC value. (**b**) Simulated and measured plating current programmability.

**Figure 16:**
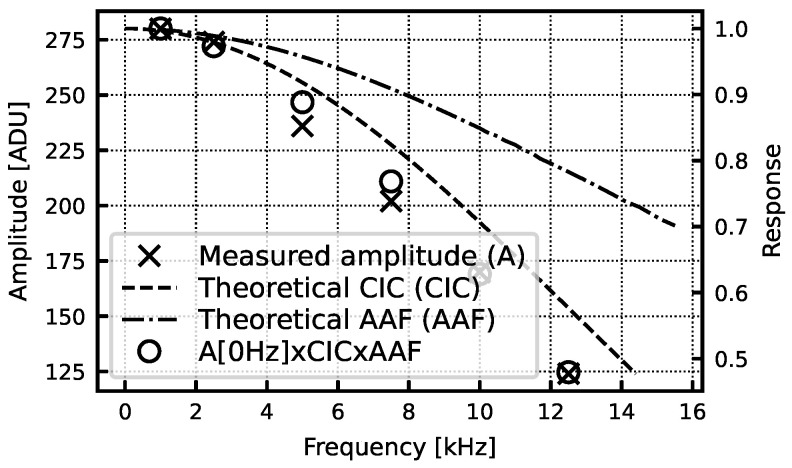
Measured amplitude for a 0.2 mV input sinusoid. The AFE gain setting is 100. ADU size is 61 µV.

Figure 15a shows measurements of the read-back analog biasing current compared to simulated values. Measurement reveals currents slightly higher than expected but still within the desired range. The plating current DAC was also measured to be comparable with the simulation results (Figure 15b). Figure 16 shows the signal amplitude over frequency for a 0.2 mV input sinusoid. The response is compared to the theoretical response of the AAF and digital CIC filter with gain scaling, and it follows the expected roll-off. It also reveals a 15% lower gain compared to the expected value in this particular gain setting.

**Figure 17:**
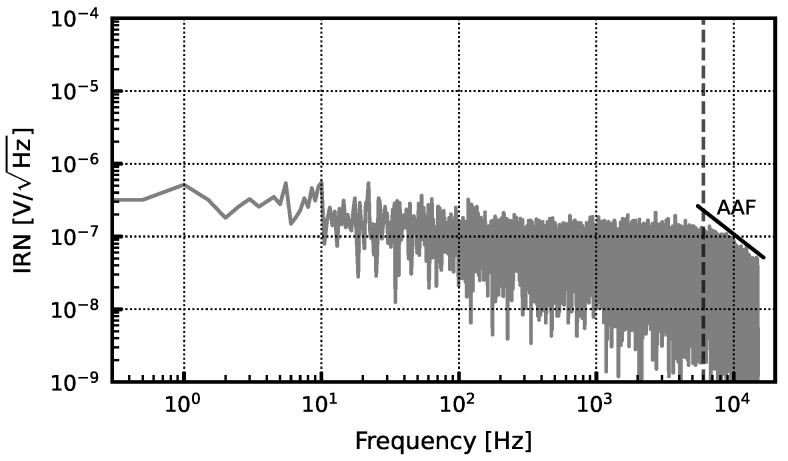
Measured noise spectrum in AP band of interest (0.3–6 kHz).

Figure 17 shows the input-referred noise (IRN) histogram. Measured noise is 5.4 µV in the 0.3–10 kHz action potential (AP) band and 3.1 µV in the 0.5 Hz–1 kHz local-field potential (LFP) band. For our application, the AP band of interest is the 0.3–6 kHz band, which yields an IRN of 4.8 µV.

**Figure 18:**
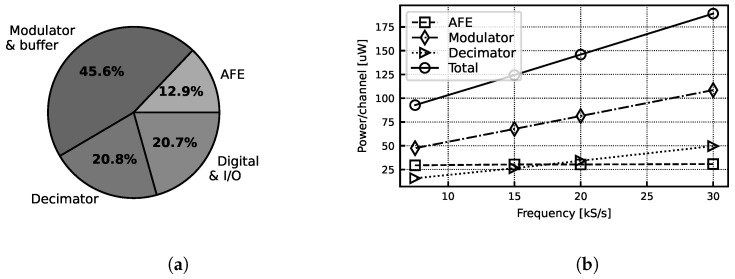
(**a**) Measured power breakdown. (**b**) Measured power at various sampling frequencies.

At a 1.8 V supply voltage, the total power consumption is 244 µW/channel when sampling at the maximum sampling frequency of 30 kS/s. This includes all on-chip components—the AFE, buffer and complete ADC—as well as all programmability features, digital communication protocol implementation and LVDS I/O circuitry. The power breakdown of the chip is shown in Figure 18.

#### 3.1.2. Data Post-Processing

Larger than anticipated leakage currents cause the offset compensation capacitor to charge up, imposing a sawtooth-shaped artifact on the signal. The primary source of this leakage is the gate leakage of the capacitor. To remove this sawtooth background, we take advantage of the consistent shape of the leakage current-induced patterns. This involves (1) detecting the up and down-phases of the sawtooth signal, (2) fitting a line to the up-phases and (3) subtracting away the linear fit while zeroing out changes in the negligibly short down-phases. Let the acquired signal be denoted *x*, *n* be the index of the discretely sampled time series, and *d* be a sampling-rate dependent delay parameter. In step 1, the down-phase of the sawtooth is detected when both a delayed amplitude threshold (1) and a first-order difference threshold (2) are met.

(2) $$x\left[n-d\right]>x_{min}$$

(3) $$\Delta x\left[n\right]<\Delta x_{max}$$

Since the leakage current is always in one direction, the phase detection criteria assumes that the sawtooth signal always rises positively; however, this can easily be generalized to sawtooth signals of the opposite sign. Samples not detected as down-phases are classified as up-phases. A least squares linear model is fit to the up-phases of *x*, ignoring the down-phases by concatenating only the up-phases of the signal *x* together. Let *c* be the slope of the linear fit. The final step involves subtracting away the linear sawtooth component of the signal, which can be achieved with the first-order difference signal and accumulating Δx[n]:

(4) $$\Delta x\left[n\right]\leftarrow \left\{\begin{array}{cc} \Delta x[n]-c & \mathrm{if}\;up-phase\\ 0 & \mathrm{if}\;down-phase \end{array}\right.$$

**Figure 19:**
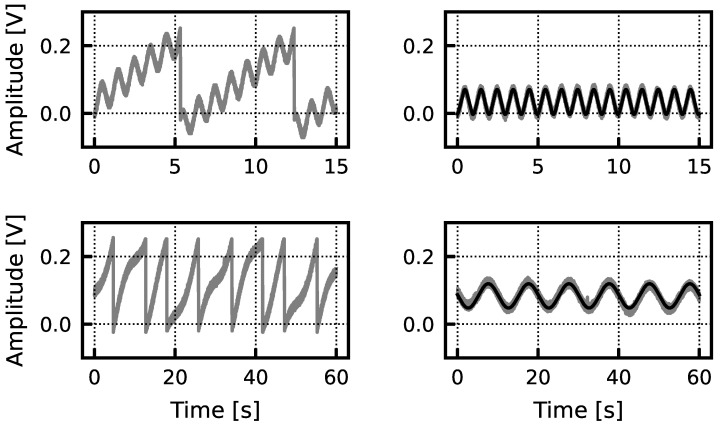
The top left panel shows raw data collected with a 1 Hz sine wave injected into the test chip. The top right shows the signal and fitted sinusoid after sawtooth removal. The bottom left panel shows raw data collected with a 0.1 Hz signal injected into the test chip. The bottom right panel shows the signal and fitted sinusoid after sawtooth removal. In both the 1 Hz and 0.1 Hz case, there is excellent agreement ($R^{2}=0.96$ and $R^{2}=0.94$ for the 1 and 0.1 Hz, respectively) between the injected sinusoids and the signals after post hoc sawtooth removal.

As shown in Figure 19, post-processing eliminates the sawtooth background in signals of frequencies both above and below the sawtooth frequency.

#### 3.1.3. Electroplating and Electrode Impedance Measurements

**Figure 20:**
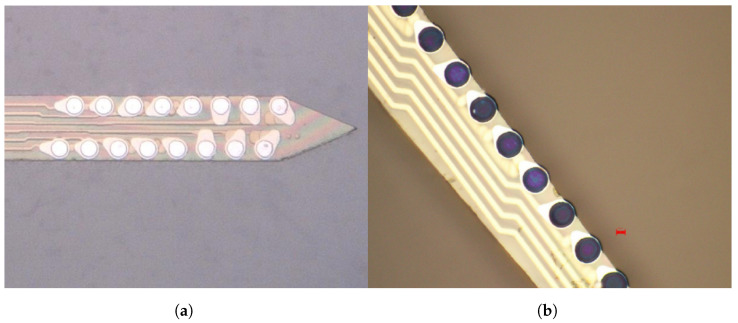
(**a**) Bare metal electrodes. (**b**) PEDOT:PSS plated electrodes.

**Figure 21:**
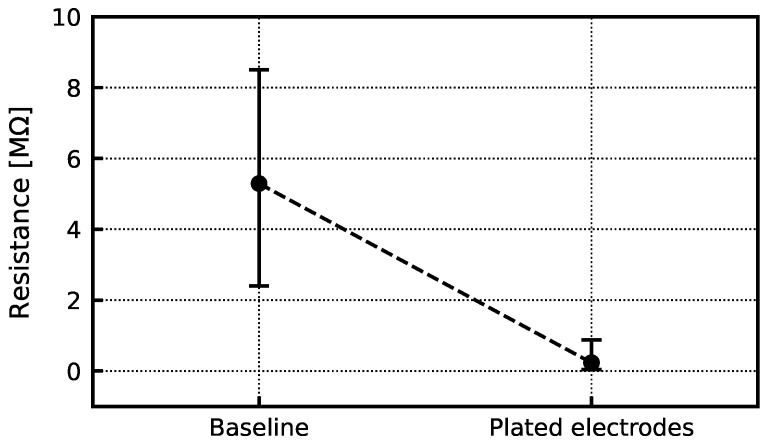
Impedance of a baseline and a PEDOT:PSS plated set of electrodes. The impedance decreases from 5.4 (90% CI = 2.4 to 8.5) to 0.2 (90% CI = 0.04 to 0.88) MΩ after electrode plating. The impedance was measured at 1 kHz.

As outlined in Section 2.2.1, the AFE is equipped with a digitally controlled electroplating DAC. There are two main steps in the electroplating process: (1) electrochemically cleaning the metal surface of the electrodes and (2) coating with poly(3,4-ethylenedioxythiophene) polystyrene sulfonate (PEDOT:PSS). Both steps are performed using a two-electrode configuration where the reference/ground electrode is a pure silver wire and the microelectrodes are 20 µm diameter platinum contacts. Cleaning involves submerging the electrodes in a sulfuric acid bath and passing a current that is swept between −30 and 150 nA at an average rate of 2 nA/s for 10 min. After cleaning, the electrode array is rinsed with 70% alcohol and deionized water. Subsequently, the electrode array is submerged in a solution of PEDOT:PSS. Finally, a constant current of 10 nA is supplied for 45 s through each electrode, simultaneously plating all electrodes. Figure 20 shows the bare metal electrodes and the plated PEDOT:PSS electrodes. As a result of plating, the impedance decreased from 5.4 (90% confidence interval [CI] = 2.4 to 8.5) to 0.2 (90% CI = 0.04 to 0.88) MΩ, as shown in Figure 21.

**Figure 22:**
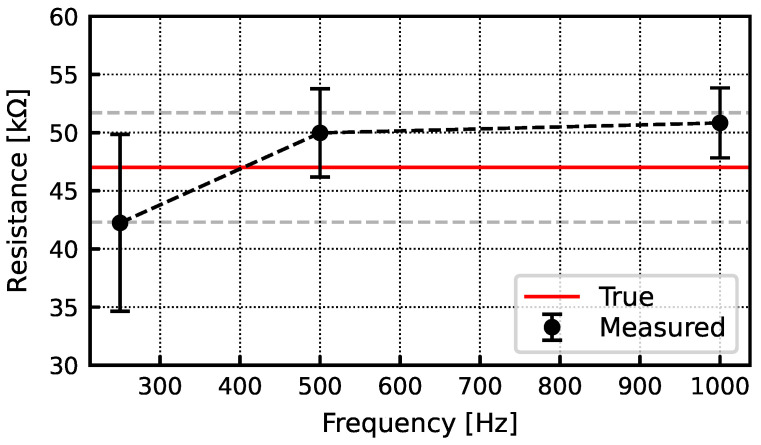
Resistance measurements using a 47 kΩ resistor. The measured resistance is within 10% of the reference.

Finally, the chip has the ability to perform electrode impedance measurements at arbitrary frequencies. Monitoring electrode impedance can provide valuable information about the quality of the recorded signal [18]. In this work, each channel can individually be programmed to enable a positive or negative plating current. By continuously re-programming the DAC that sets the plating current for the chip, current patterns can be implemented including sinusoids. Electrode impedance can be measured by passing a sinusoidal current through an electrode and measuring the voltage across the electrode. In a sample of six channels, the resistance measured across a 47 kΩ resistor at 250, 500, and 1000 Hz was ≤±10%, as shown in Figure 22.

### 3.2. In Vivo Electrophysiology

**Figure 23:**
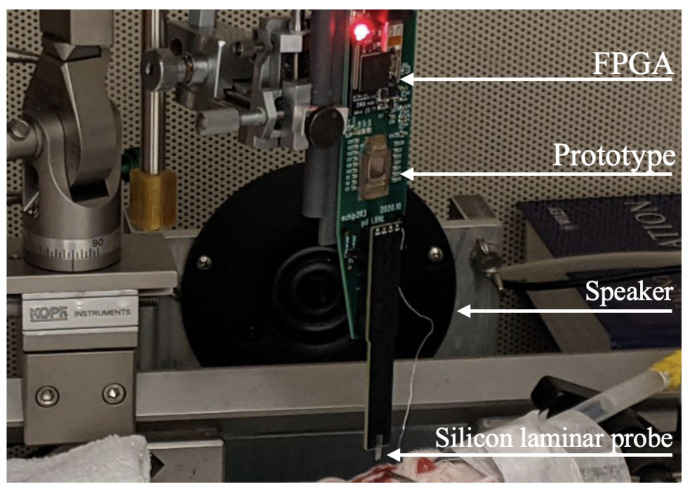
Photo of the in vivo test setup. A custom testboard was made in order to interface the chip prototype with the commercial silicon laminar probe. Auditory stimulus was provided through a speaker, and results were post-processed using an FPGA.

We performed an acute craniotomy experiment on a Sprague–Dawley rat, which was anesthetized by intraperitoneal injections of ketamine and xylazine. All rat procedures were performed in accordance with established animal care protocols approved by the LBNL Institutional Animal Care and Use Committees (IACUC). A commercial silicon laminar probe was inserted into the primary auditory cortex, and a platinum reference wire was inserted into a contralateral frontal region. A testboard was fabricated to connect the silicon laminar probe with the chip. Figure 23 shows a photo on the test setup, including the custom testboard, prototype, and silicon laminar probe.

The auditory stimulus included a white noise burst lasting 100 ms played every 1 s for 60 repetitions. The digital output was sent to an FPGA and main controller unit for digital processing and finally sent to a computer for visualization and data saving (FPGA development and post-processing was performed by our collaborators at SpikeGadgets, San Francisco, CA, USA).

The recordings were post-processed with a similar background subtraction technique described in Section 3.1.2 and then passed through spectral and spike-sorting analysis pipelines. The spectral analysis involves computing the constant-Q wavelet transform for each trial for center frequencies ranging from 8.3 to 1200 Hz [19]. The magnitude of the transform is taken and then normalized by z-scoring relative to the baseline. The baseline period lasts 200 samples or ∼6.67 ms and starts 100 ms prior to the upcoming stimulus presentation.

For a separate spike analysis, high-pass filtering at 300 Hz, whitening, and automated spike clustering were performed using the publicly available spike-sorting algorithms spikeinterface [20] and MountainSort [10]. Finally, the produced units or clusters were manually curated to identify putative single units.

**Figure 24:**
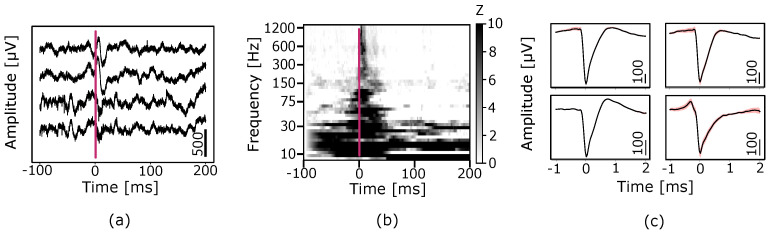
Chip readout of in vivo electrophysiological signals. (**a**) High-pass filtered ($f_{c}$ = 300 Hz) signals from 4 channels during the presentation of an auditory stimulus indicated by the red vertical line at time 0. (**b**) Median spectrogram across 60 trials of auditory presentation showing a broadband increase in amplitude relative to a baseline window. The auditory presentation is indicated by the red vertical line at time 0. The amplitude is normalized by z−scoring (Z) relative to baseline. (**c**) Four putative single unit waveforms generated by using an automated spike−sorting algorithm. The average waveform is plotted in black, and the 95% standard error is plotted as a red-shaded region about the average.

The prototype was able to readout in vivo electrophysiological signals including action potentials measured from laminar polytrodes inserted into a cortex. The filtered measured signals from four channels are shown in Figure 24a. Evoked potentials were strongly driven by auditory stimuli across the neural frequency spectrum as expected (Figure 24b). Spike sorting revealed isolated putative single units (Figure 24c). These results demonstrate that the proposed design can readout spikes and field potentials that are modulated by a sensory input.

The chip performance summary is shown in Table 2 compared to the two most widely adopted state-of-art commercial neural signal acquisition systems.

## 4. Conclusions

We report a massive 512-channel neural signal acquisition ASIC designed to target high-density electrophysiology. Modularity and scalability enable addressing mutliple brain regions and were key components of the design as well as integration with commercial high-density probe systems. We briefly discuss our complete system headstage design which targets 4096-channel recording and focus on the chip design, testing, and data processing. The ASIC features programmable gain, filtering, and 14b ΣΔ digitization, including digital decimation filtering in each channel. It occupies a 55.8 mm^2^ area, measures an IRN of 4.8 µV in the AP band of interest (0.3–6 kHz), and dissipates 244 µW/channel from a 1.8 V supply. The chip also provides electrode plating and electrode impedance measurement capability. Finally, we present in vivo measurements of action potentials using silicon laminar probes on anesthetized rats. This work demonstrates an ultra-low-noise flexible signal acquisition modular system with potential for ultra-high-density neural recording.

## Figures and Tables

**Table 1:** Transistor usage in implemented CIC filter.

Circuit	Instances	Transistors	Total Transistors
Coder	1	42	42
Integrator	3	880	2640
Downsampler	1	132	132
Differentiator	3	1012	3036
Complete Filter	1	5850	5850

**Table 2:** Performance summary.

	[5]	[6]	[13]	[21]	This Work
Channels	64	384	128	16	512
Tot. area [mm^2^]	28.7	45.2	0.005	5.8	55.8
Area/ch. [mm^2^]	0.448 ^a^	0.12	0.0045	0.16	0.099
ADC bits	16	10	14	8	14
ADC $f_{s}$ [kHz]	30	30	30	31.25	30
IRN (LFP) [μV]	2.4 ^b^	10.32	11.9	-	3.1
IRN (AP) [µV]	2.4 ^b^	6.36	7.71	5.4 ^c^	5.4
Power/ch. [µW]	351	49.06	8.34	0.96	244
Supply [V]	3.0	1.2/1.8	0.8	0.5	1.8
In vivo results	Yes	Yes	No	Yes	Yes
Technology [µm]	0.35	0.13 SOI	0.022	0.18	0.18

^a^ Includes I/O and digital interface. ^b^ Unspecified frequency range. ^c^ 1–12 kHz frequency range.

## Data Availability

No new data were created or analyzed in this study. Data sharing is not applicable to this article.
